# Hepatorenal dysfunction in patients with chronic thromboembolic pulmonary hypertension

**DOI:** 10.3389/fmed.2023.1207474

**Published:** 2023-07-20

**Authors:** Athiththan Yogeswaran, Daniel Zedler, Manuel J. Richter, Sonja Steinke, Zvonimir A. Rako, Nils C. Kremer, Friedrich Grimminger, Werner Seeger, Hossein Ardeschir Ghofrani, Henning Gall, Khodr Tello

**Affiliations:** ^1^Department of Internal Medicine, Member of the German Center for Lung Research (DZL), Justus-Liebig-University Giessen, Universities of Giessen and Marburg Lung Center (UGMLC), Giessen, Germany; ^2^Department of Internal Medicine, Member of the German Center for Lung Research (DZL), Institute for Lung Health (ILH), Cardio-Pulmonary Institute (CPI), Universities of Giessen and Marburg Lung Center (UGMLC), Giessen, Germany

**Keywords:** pulmonary hypertension, chronic thromboembolic pulmonary hypertension, hepatorenal function, echocardiography, strain

## Abstract

**Background:**

Cardiac interactions with organs such as the liver or kidneys have been described in different cardiovascular diseases. However, the clinical relevance of hepatorenal dysfunction in chronic thromboembolic pulmonary hypertension (CTEPH) remains unclear. We determined the association of hepatorenal dysfunction (measured using the Model for End-stage Liver Disease Sodium [MELDNa] score) with right heart function and survival in patients with CTEPH.

**Methods:**

We analyzed all patients with CTEPH in the Giessen Pulmonary Hypertension Registry who had available MELDNa scores and were not taking vitamin K antagonists. The MELDNa score was calculated as MELD score − serum Na − (0.025 * MELD score * (140 − serum Na)) + 140; the MELD score was calculated as 10*(0.957*ln(creatinine)+0.378*ln(bilirubin)+1.12*ln(International Normalized Ratio))+6.43.

**Results:**

Seventy-two patients were included (74% female; median [Q1, Q3] MELDNa: 9 [6, 11]). MELDNa correlated well with right atrial and ventricular function and pulmonary hemodynamics. Forward regression analysis revealed that hepatorenal dysfunction mainly depends on right atrial strain and tricuspid regurgitation, but not right ventricular systolic dysfunction. Hepatorenal dysfunction predicted mortality at baseline and follow-up (adjusted hazard ratios [95% confidence intervals] per unit increase of MELDNa: 1.6 [1.1, 2.4] and 1.8 [1.1, 2.9], respectively). Changes in hepatorenal function also predicted mortality.

**Conclusion:**

Hepatorenal dysfunction in CTEPH is primarily associated with venous congestion rather than cardiac forward failure. As a surrogate parameter for hepatorenal dysfunction, MELDNa is a simple method to identify at-risk patients at baseline and follow-up.

## 1. Introduction

Chronic thromboembolic pulmonary hypertension (CTEPH) is a progressive and life-threatening disease that ultimately leads to right heart insufficiency and premature death. Owing to the pathophysiological nature of the disease (which is characterized by pulmonary artery obstructions), right ventricular (RV) afterload and wall tension are increased, and the right ventricle seeks to counteract these changes with cardiac remodeling. First, homeometric adaptation according to the Laplace law leads to concentric hypertrophy and increased wall thickness. A further increase in RV afterload can then lead to heterometric (mal)adaptation with right heart dilatation, RV insufficiency, and venous congestion (1).

Clinical studies have focused on the right ventricle and its systolic (dys)function (2–4). However, an increase in RV stiffness can also lead to diastolic dysfunction, even before RV systolic failure is obvious. RV diastolic dysfunction may cause vena cava backflow during contraction of the right atrium and tricuspid regurgitation (TR) (5). It is well known that the resulting venous congestion, as well as the forward failure of the right ventricle, can cause organ dysfunction, which is inextricably linked to disease progression (6, 7).

The liver and kidneys are affected by both venous congestion and reduced cardiac output (8, 9). There are various methods for assessing hepatorenal dysfunction, for example liver and kidney sonography, which we and others have proven to be feasible and highly relevant for patients with pulmonary hypertension (PH) (10, 11). A more simple yet comprehensive method for assessing hepatorenal dysfunction is based on the Model for End-stage Liver Disease Sodium (MELDNa) score (12–14). The MELDNa score is usually used for patients with liver cirrhosis or end-stage liver disease, particularly for organ allocation, but its prognostic and clinical relevance has also been shown in patients with various other diseases, including left ventricular failure, primary TR, and congenital heart disease (6, 15). In addition, the MELDNa score is easily available in clinical routine as it is based on commonly measured laboratory parameters.

It is, however, unclear whether the non-invasive assessment of hepatorenal dysfunction is also associated with the severity of PH and whether the MELDNa score is prognostically relevant for patients with CTEPH. Furthermore, the underlying pathomechanism leading to hepatorenal dysfunction in patients with CTEPH is not fully understood. Therefore, the aim of this study was to determine the association of hepatorenal dysfunction (measured using the MELDNa score) with right heart function and survival in patients with CTEPH.

## 2. Materials and methods

### 2.1. Study design and patients

All patients with confirmed CTEPH enrolled between June 2013 and January 2021 in the Giessen PH Registry (16) were included in this retrospective study. The diagnosis was made by a multidisciplinary board of physicians, radiologists, and surgeons according to the contemporary guidelines (17). The date of the diagnostic right heart catheterization was defined as the date of diagnosis. Patients with missing data for calculation of the MELDNa score and patients taking vitamin K antagonists were excluded from the primary analyses. Patients with missing data for calculation of the MELD excluding international normalized ratio (INR) score (defined below) were excluded from the sensitivity analyses. All patients were followed up until November 2022.

The study was approved by the Institutional Review Board of the University of Giessen (No. 266/11), and all patients gave their written informed consent.

### 2.2. Right heart echocardiography and catheterization

Right heart echocardiography and catheterization were performed as described previously (18, 19). In brief, all echocardiographic parameters were obtained according to the current guidelines (20) *via* a GE Vivid E9 ultrasound unit (GE Healthcare, Wauwatosa, Wisconsin, United States). Tricuspid annular plane systolic excursion (TAPSE) was measured in M-mode at the lateral tricuspid valve annulus. Right atrial (RA) area was measured at end-systole corresponding to the maximal area. RA pressure (RAP) was estimated from the diameter of the inferior vena cava and its inspiratory collapsibility (17). Pulmonary arterial systolic pressure was estimated by adding estimated RAP to the tricuspid valve gradient (21). The severity of TR was categorized into three levels as previously described (22). Right heart speckle-tracking echocardiography was performed according to the consensus document of the European Association of Cardiovascular Imaging/American Society of Echocardiography/Industry Task Force (23).

All echocardiographic parameters that were not routinely acquired were obtained by an independent investigator who was blinded to the clinical data. Echocardiographic measurements and analyses were performed with EchoPac software (version 201, GE Healthcare).

### 2.3. Assessment of hepatorenal dysfunction

The surrogate parameter for hepatorenal dysfunction used in this paper was the MELDNa score, which adds sodium levels to the original MELD score (12). The MELDNa score was calculated as MELD score − serum Na − (0.025 * MELD score * (140 − serum Na)) + 140, while the MELD score was calculated as 10*(0.957*ln(creatinine)+0.378*ln(bilirubin)+1.12* ln(International Normalized Ratio))+6.43. Hepatorenal dysfunction was defined as a MELDNa score above the third quartile (> 11).

In sensitivity analyses, hepatorenal dysfunction was defined as a MELD excluding INR (MELD-XI) score (24) above the third quartile (≥ 12 at baseline and ≥ 11 during follow-up). The MELD-XI score, which has been associated with prognosis in patients with acute heart failure (25), was calculated as 5.11 x ln(bilirubin) + 11.76 x ln(creatinine) + 9.44, as described by Heuman et al. (24).

### 2.4. Statistical analyses

Statistical analyses were performed using R version 4.2.1 (The R Foundation for Statistical Computing) and SPSS version 29 (IBM, Armonk, United States). All parameters were evaluated based on the Shapiro–Wilk normality test and histograms for normal distribution. When normally distributed, data are presented as the mean (standard deviation) and compared using Student’s t-tests. Otherwise, numbers are presented as median [Q1, Q3] and compared using Wilcoxon rank tests. The linear association between variables was assessed using Spearman’s rho. Furthermore, we used univariate and multivariate binary regression analyses to investigate the influence of different parameters on hepatorenal dysfunction in patients with CTEPH. In addition, survival analyses were performed using Kaplan–Meier estimators as well as univariate and multivariate Cox regression.

## 3. Results

### 3.1. Study population

In total, 117 patients with CTEPH had available MELD-XI scores (screening population; Supplementary Table 1), of whom 111 also had available MELDNa scores (Supplementary Table 2). Of the 111 patients, 39 were excluded from the primary analyses because they used vitamin K antagonists. Seventy-two patients remained and were included in the primary analyses; 74% were female and the median age was 71 [56, 78] years. Baseline characteristics of the study cohort indicated a marked impairment of pulmonary hemodynamics, as shown in Table 1. In addition, the majority of the included patients (64%) showed at least moderate TR. Further echocardiographic and laboratory parameters are shown in Table 1. The median MELDNa score was 8.95 [6.36, 11.06].

**Table 1:** Baseline characteristics.

	*n*	Preserved hepatorenal function^*^ (*n* = 53)	Hepatorenal dysfunction^*^ (*n* = 19)	Total (*n* = 72)	*p*
Age, years	72	74.0 [57.0, 78.0]	68.0 [55.0, 75.5]	71.0 [55.8, 78.0]	0.338^†^
Male sex, *n* (%)	72	18 (34)	8 (42)	26 (36)	0.526^‡^
Body mass index, kg/m^2^	72	27.61 [25.40, 30.53]	28.48 [25.08, 32.15]	27.89 [25.39, 30.82]	0.845^†^
NYHA FC, *n* (%):	72				0.573^‡^
1		0	1 (5)	1 (1)	
2		6 (11)	2 (11)	8 (11)	
3		23 (43)	8 (42)	31 (43)	
4		4 (8)	1 (5)	5 (7)	
NA		20 (38)	7 (37)	27 (38)	
mPAP, mm Hg	72	36.0 [28.0, 47.0]	46.0 [40.0, 52.0]	38.0 [31.0, 47.2]	0.013^†^
PAWP, mm Hg	72	10.00 [8.00, 13.00]	10.00 [7.50, 16.00]	10.00 [8.00, 13.25]	0.805^†^
RAP, mm Hg	72	7.00 [5.00, 10.00]	10.00 [8.00, 13.00]	8.00 [5.75, 11.00]	0.009^†^
Cardiac output, L/min^§^	72	4.43 ± 1.14	3.59 ± 1.13	4.21 ± 1.19	0.009
PVR, dyn·s/cm^5^	72	400 [251, 720]	629 [504, 896]	537 [269, 822]	0.014^†^
Cardiac index, L/min/m^2^	72	2.529 ± 0.575	2.115 ± 0.388	2.420 ± 0.560	0.001
SvO_2_, %	72	65.60 [60.40, 69.80]	55.40 [50.80, 62.20]	63.00 [58.45, 68.73]	<0.001^†^
INR, *n* (%)	72	1.0 [1.0, 1.1]	1.2 [1.1, 1.3]	1.1 [1.0, 1.2]	0.003^†^
Sodium, mEq/L	72	140.00 [139.00, 142.00]	138.00 [137.00, 140.00]	140.00 [138.00, 141.25]	<0.001^†^
Creatinine, mg/dL	72	0.900 [0.800, 1.100]	1.200 [0.950, 1.300]	0.900 [0.800, 1.200]	0.009^†^
Estimated GFR, mL/min/1.73 m^2^	72	74.1 [62.9, 88.2]	57.6 [46.6, 73.6]	70.2 [51.3, 88.0]	0.045^†^
Albumin, g/L	71	41.65 [38.20, 43.52]	39.70 [36.85, 42.80]	41.30 [37.20, 43.50]	0.196^†^
Total bilirubin, mg/dL	72	0.600 [0.400, 0.900]	1.000 [0.600, 1.550]	0.700 [0.475, 1.000]	0.006^†^
BNP, pg./mL	70	147.0 [57.5, 253.5]	511.0 [242.5, 925.5]	183.0 [74.2, 363.5]	<0.001^†^
TAPSE, mm	69	20.83 ± 3.81	17.32 ± 4.23	19.96 ± 4.17	0.005
S′, cm/s	63	12.00 [10.0, 15.00]	11.00 [9.00, 13.00]	12.00 [10.0, 15.00]	0.145^†^
TR severity, *n* (%):	72				0.195^‡^
None		2 (4)	1 (5)	3 (4)	
Mild		18 (34)	2 (11)	20 (28)	
Moderate		21 (40)	8 (42)	29 (40)	
Severe		11 (21)	6 (32)	17 (24)	
NA		1 (2)	2 (11)	3 (4)	
IVC diameter on expiration, mm	52	19.50 [15.00, 22.00]	20.00 [17.00, 22.00]	20.00 [16.00, 22.00]	0.493^†^
RA reservoir strain, %	53	28.0 [18.6, 33.7]	14.8 [12.1, 19.5]	23.1 [15.2, 31.9]	<0.001^†^
RA conduit strain, %	52	8.75 [3.94, 13.41]	5.00 [3.12, 6.45]	6.45 [3.48, 13.03]	0.067^†^
RA active strain, %	49	17.53 [12.55, 22.27]	10.67 [8.44, 14.53]	15.13 [10.78, 20.16]	0.004^†^
RV GLS, %	40	−16.60 [−19.43, −10.18]	−11.00 [−15.22, −8.45]	−16.00 [−17.80, −8.85]	0.041^†^
RA area, cm^2^	62	17.39 ± 6.94	19.54 ± 5.49	18.02 ± 6.58	0.20
RV EDD, cm	63	4.667 ± 0.823	5.011 ± 0.601	4.766 ± 0.777	0.074
MELDNa score	72	7.44 ± 2.21	13.04 ± 1.28	8.92 ± 3.19	<0.001
		7.76 [5.80, 9.20]	13.00 [12.15, 13.60]	8.95 [6.36, 11.06]	
DOAC, *n* (%)	72	44 (83)	17 (89)	61 (85)	0.502^‡^

* Hepatorenal dysfunction was defined as a MELDNa score above the third quartile (> 11).

† Wilcoxon test.

‡ Pearson test.

§ Measured by thermodilution.

Data are presented as mean ± standard deviation, median [interquartile range], or *n* (%). *p* values are from *t*-tests unless otherwise specified. BNP, B-type natriuretic peptide; DOAC, direct oral anticoagulant; EDD, end-diastolic diameter; GFR, glomerular filtration rate; GLS, global longitudinal strain; INR, international normalized ratio; IVC, inferior vena cava; MELDNa, model for end-stage liver disease sodium; mPAP, mean pulmonary arterial pressure; NA, not available; NYHA FC, New York Heart Association functional class; PAWP, pulmonary arterial wedge pressure; PVR, pulmonary vascular resistance; RA, right atrial; RAP, right atrial pressure; RV, right ventricular; SvO_2_, mixed venous oxygen saturation; TAPSE, tricuspid annular plane systolic excursion; TR, tricuspid regurgitation.

### 3.2. Association of hepatorenal dysfunction with right heart parameters in CTEPH

Correlation analyses indicated that the MELDNa score has a linear association with RA and RV function (Table 2). In particular, the MELDNa score was associated with RA reservoir strain (rho: − 0.515, *p* < 0.001), RV end-systolic area (rho: 0.408, *p* = 0.004), and TAPSE (rho: − 0.390, *p* < 0.001). TR severity (rho: 0.298, *p* = 0.013) and RA area (rho: 0.273, *p* = 0.032) were also significantly linked to hepatorenal (dys)function. Left ventricular global longitudinal strain (GLS), however, was not associated with the MELDNa score (*p* = 0.532).

**Table 2:** Correlation of MELDNa with RA and RV parameters and pulmonary hemodynamics.

Parameter	*R*	*p*
mPAP	0.312	0.008
PAWP	−0.037	0.755
RAP	0.091	0.448
Cardiac output (thermodilution)	−0.312	0.008
PVR	0.349	0.003
Cardiac index	−0.365	0.002
sPAP	0.312	0.008
dPAP	0.298	0.011
SvO_2_	−0.460	<0.001
BNP	0.492	<0.001
TAPSE	−0.390	<0.001
TAPSE/PASP	−0.338	0.001
RA reservoir strain	−0.515	<0.001
RA active strain	−0.404	0.004
RA conduit strain	−0.326	0.018
RV GLS	0.362	0.022
LA reservoir strain	−0.447	0.017
LV GLS	0.175	0.532
RA area	0.273	0.032
RV EDD	0.307	0.014
RV end-systolic area	0.408	0.004
RV end-diastolic area	0.381	0.007
TR severity	0.298	0.013

BNP, B-type natriuretic peptide; dPAP, diastolic pulmonary arterial pressure; EDD, end-diastolic diameter; GLS, global longitudinal strain; LA, left atrial; LV, left ventricular; MELDNa, Model for End-stage Liver Disease Sodium; mPAP, mean pulmonary arterial pressure; PAWP, pulmonary arterial wedge pressure; PVR, pulmonary vascular resistance; RA, right atrial; RAP, right atrial pressure; RV, right ventricular; sPAP, systolic pulmonary arterial pressure; SvO_2_, mixed venous oxygen saturation; TAPSE, tricuspid annular plane systolic excursion; PASP, pulmonary artery systolic pressure; TR, tricuspid regurgitation.

The parameters most stringently related to the MELDNa score were ascertained by multivariate linear regression analysis using the forward selection technique The following parameters were included: systolic, diastolic, and mean pulmonary arterial pressure, pulmonary vascular resistance, cardiac index, sex, TR severity, TAPSE, RA reservoir strain, RV GLS, RA area, RV diameter, RV end-systolic area, and RAP. Interestingly, the model showed that RA reservoir strain (*β*-coefficient: − 0.365, *p* = 0.003) and TR severity (*β*-coefficient: 0.385, *p* = 0.002), but not RV systolic (dys)function, were strongly related to hepatorenal (dys)function.

The study cohort was then divided into two groups based on the MELDNa score. Patients with a high MELDNa score (above the third quartile [> 11]) were defined as patients with hepatorenal dysfunction. Patients fulfilling this definition of hepatorenal dysfunction had more severely impaired pulmonary hemodynamics (Table 1) and significantly higher B-type natriuretic peptide (BNP) concentrations (511.0 [242.5, 925.5] pg./mL vs. 147.0 [57.5, 253.5] pg./mL, *p* < 0.001) than those without hepatorenal dysfunction. Furthermore, RV and RA strain were more severely impaired (TAPSE: 17.32 ± 4.23 mm vs. 20.83 ± 3.81 mm, *p* = 0.005; RV GLS: − 11.00 [− 15.22, − 8.45]% vs. − 16.60 [−19.43, −10.18]%, *p* = 0.041; RA reservoir strain: 14.8 [12.1, 19.5]% vs. 28.0 [18.6, 33.7]%, *p* < 0.001). No other significant differences between the two groups were detected (Table 1).

### 3.3. Prognostic relevance of hepatorenal dysfunction in CTEPH

**Figure 1:**
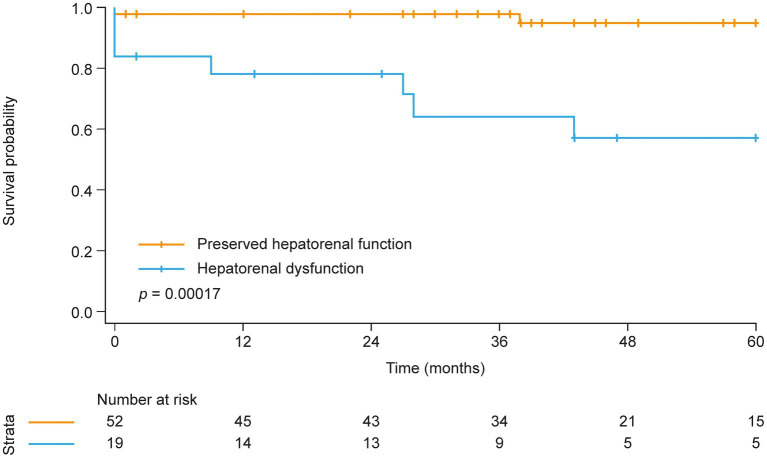
Kaplan–Meier analysis of survival in patients with and without hepatorenal dysfunction at baseline.

Next, Kaplan–Meier analysis was performed to investigate the prognostic relevance of hepatorenal dysfunction in patients with CTEPH (Figure 1). Survival at 1, 3, and 5 years was 98, 98, and 95%, respectively, in patients with preserved hepatorenal function but significantly lower in patients with hepatorenal dysfunction (79, 64, and 57%, respectively). Univariate Cox regression showed a significantly increased hazard ratio (HR) per unit increase of MELDNa (HR: 1.6 [95% confidence interval (CI): 1.2, 2.1], *p* = 0.001). Hepatorenal dysfunction remained as an independent predictor of mortality even after adjusting for age, RV systolic function (TAPSE), and TR severity (HR: 1.6 [95% CI: 1.1, 2.3], *p* = 0.017). Further adjustment for direct oral anticoagulant (DOAC) intake did not change the results (HR: 1.6 [95% CI: 1.1, 2.4], *p* = 0.019).

A sensitivity analysis of the screening population (*n* = 117) stratified by the MELD-XI score (24) also showed impaired survival in patients with hepatorenal dysfunction at baseline (Supplementary Figure 1A; HR: 3.67 [95% CI: 1.55, 8.65], *p* = 0.003).

### 3.4. Patient characteristics and clinical and prognostic relevance of hepatorenal dysfunction at follow-up

Follow-up data were available in 58 cases in the study cohort. The median time interval between diagnosis and follow-up was 6 [3, 9] months. Characteristics of the patients at follow-up are shown in Table 3. During the follow-up period, 15 (26%) and 2 (3%) patients underwent pulmonary endarterectomy and balloon pulmonary angioplasty, respectively, while PH-targeted medication was initiated in 38 patients (66%).

**Table 3:** Follow-up characteristics.

	*n*	Preserved hepatorenal function^*^ (*n* = 43)	Hepatorenal dysfunction^*^ (*n* = 15)	Total (*n* = 58)	*p*
Age, years	58	73.00 [65.00, 78.50]	69.00 [64.00, 76.00]	72.00 [64.25, 78.00]	0.958^†^
Male sex, *n* (%)	58	17 (40)	5 (33)	22 (38)	0.67^†^
Body mass index, kg/m^2^	48	27.17 [25.35, 29.16]	27.37 [25.08, 31.96]	27.17 [25.21, 29.44]	0.708^†^
NYHA FC, *n* (%):	58				0.021^‡^
2		15 (35)	1 (7)	16 (28)	
3		22 (51)	7 (47)	29 (50)	
4		0	1 (7)	1 (2)	
NA		6 (14)	6 (40)	12 (21)	
mPAP, mm Hg	34	32.54 ± 10.79	40.88 ± 8.17	34.50 ± 10.74	0.034
PAWP, mm Hg	34	11.88 ± 4.58	10.12 ± 5.67	11.47 ± 4.83	0.442
RAP, mm Hg	34	6.77 ± 3.87	8.00 ± 4.93	7.06 ± 4.10	0.532
Cardiac output, L/min^§^	34	5.19 ± 1.47	4.97 ± 1.38	5.14 ± 1.43	0.714
PVR, dyn·s/cm^5^	34	253 [186, 429]	445 [338, 584]	300 [200, 461]	0.047^†^
Cardiac index, L/min/m^2^	34	2.825 ± 0.627	2.675 ± 0.532	2.789 ± 0.602	0.517
SvO_2_, %	34	65.58 ± 7.25	61.06 ± 7.71	64.52 ± 7.50	0.169
INR	58	1.000 [1.000, 1.100]	1.100 [1.050, 1.700]	1.100 [1.000, 1.100]	0.022^†^
Sodium, mEq/L	58	140.00 [139.00, 142.00]	137.00 [134.00, 138.50]	140.00 [138.00, 141.00]	<0.001^†^
Creatinine, mg/dL	58	0.900 [0.750, 1.000]	1.200 [0.950, 1.500]	0.900 [0.800, 1.100]	0.001^†^
Estimated GFR, mL/min/1.73 m^2^	58	79.1 ± 23.2	64.3 ± 47.6	75.3 ± 31.5	0.264
Albumin, g/L	58	42.40 [40.45, 44.35]	40.90 [32.15, 43.95]	41.85 [39.73, 44.08]	0.117^†^
Total bilirubin, mg/dL	58	0.600 [0.400, 0.750]	0.700 [0.500, 1.150]	0.600 [0.400, 0.800]	0.185^†^
BNP, pg./mL	55	104.0 [57.8, 174.0]	372.0 [83.0, 731.0]	116.0 [74.5, 298.5]	0.012^†^
TAPSE, mm	58	21.51 ± 3.48	17.90 ± 5.35	20.58 ± 4.30	0.025
S′, cm/s	55	13.17 ± 2.70	10.54 ± 2.44	12.55 ± 2.85	0.003
TR severity, *n* (%):	58				0.203^‡^
None		1 (2)	0	1 (2)	
Mild		16 (37)	2 (13)	18 (31)	
Moderate		16 (37)	7 (47)	23 (40)	
Severe		10 (23)	5 (33)	15 (26)	
NA		0	1 (7)	1 (2)	
IVC diameter on expiration, mm	47	17.82 ± 3.97	19.92 ± 5.01	18.40 ± 4.33	0.395
RA reservoir strain, %	43	28.41 [23.00, 36.00]	16.95 [13.60, 25.50]	27.00 [18.36, 35.97]	0.035^†^
RV GLS, %	33	−17.48 ± 4.09	−15.14 ± 5.47	−17.12 ± 4.31	0.346
RA area, cm^2^	53	16.25 [15.15, 18.90]	21.50 [16.50, 27.00]	16.80 [15.20, 20.80]	0.068^†^
RV EDD, cm	53	4.753 ± 0.626	5.154 ± 0.729	4.851 ± 0.668	0.509
MELDNa score	58	7.11 ± 2.68	14.75 ± 3.61	9.08 ± 4.46	
		7.50 [5.96, 9.03]	13.94 [12.68, 14.75]	8.36 [6.45, 10.99]	<0.001^†^
DOAC, *n* (%)	58	41 (95)	11 (73)	52 (90)	0.016^‡^
Riociguat, *n* (%)	58	29 (67)	9 (60)	38 (66)	0.518^‡^
BPA up to follow-up, *n* (%)	58	2 (5%)	0 (0%)	2 (3%)	0.198
PEA up to follow-up, *n* (%)	58	12 (28%)	3 (20%)	15 (26%)	0.547

* Hepatorenal dysfunction was defined as a MELDNa score above the third quartile (> 11).

† Wilcoxon test.

‡ Pearson test.

§ Measured by thermodilution.

Data are presented as mean ± standard deviation, median [interquartile range], or n (%). *p* values are from *t*-tests unless otherwise specified. BNP, B-type natriuretic peptide; BPA, balloon pulmonary angioplasty; DOAC, direct oral anticoagulant; EDD, end-diastolic diameter; GFR, glomerular filtration rate; GLS, global longitudinal strain; INR, International Normalized Ratio; IVC, inferior vena cava; MELDNa, Model for End-stage Liver Disease Sodium; mPAP, mean pulmonary arterial pressure; NA, not available; NYHA FC, New York Heart Association functional class; PAWP, pulmonary arterial wedge pressure; PEA, pulmonary endarterectomy; PVR, pulmonary vascular resistance; RA, right atrial; RAP, right atrial pressure; RV, right ventricular; SvO_2_, mixed venous oxygen saturation; TAPSE, tricuspid annular plane systolic excursion; TR, tricuspid regurgitation.

**Figure 2:**
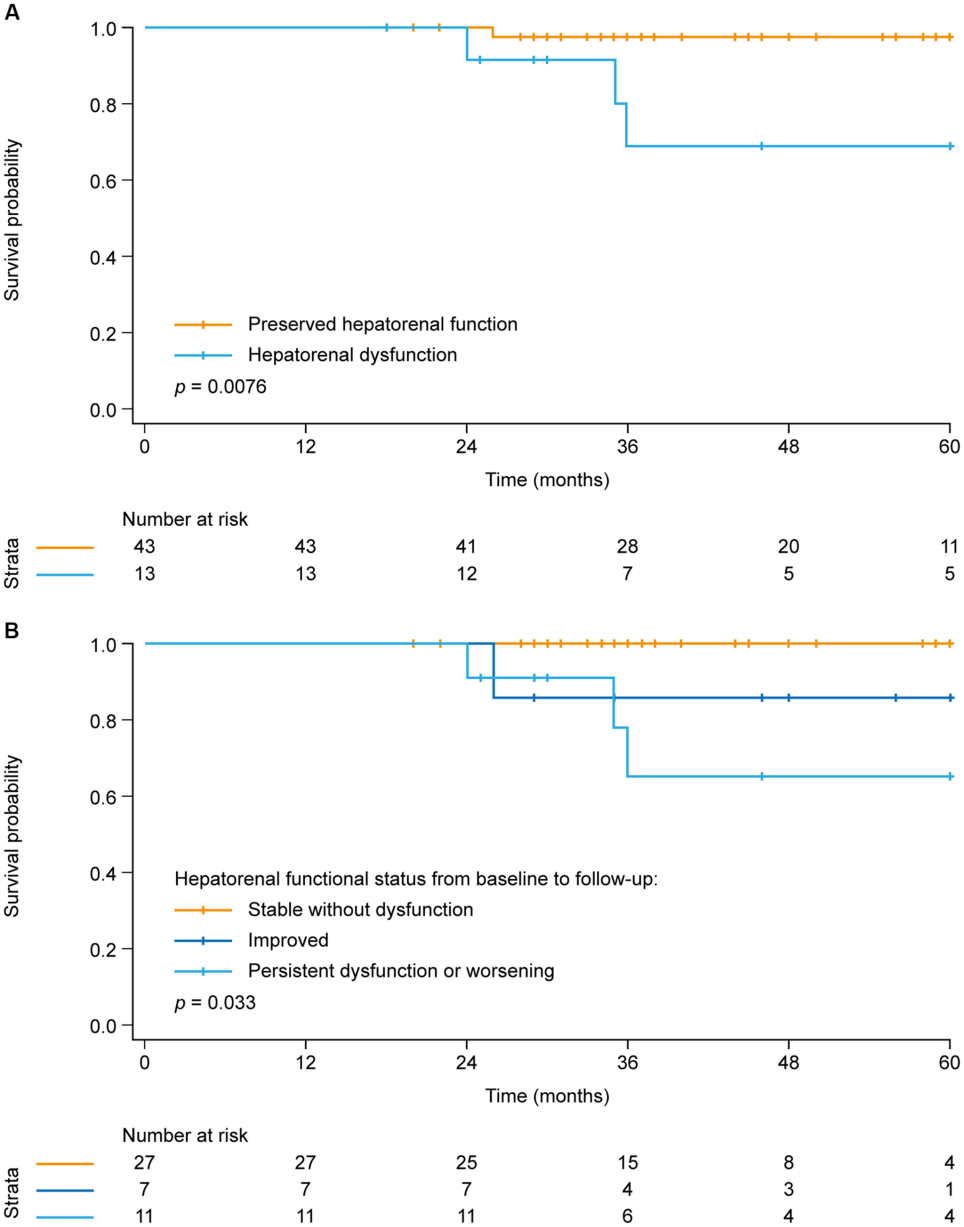
Kaplan–Meier analysis of survival in **(A)** patients with and without hepatorenal dysfunction at follow-up and **(B)** patients stratified by hepatorenal functional status change from baseline to follow-up.

Dividing the study population into the two groups with and without hepatorenal dysfunction as defined above, the analysis showed a similar interdependency of parameters as observed at baseline: Patients with hepatorenal dysfunction (MELDNa score above the third quartile [> 11]) had more severe impairment of pulmonary hemodynamics, higher BNP concentrations, and impaired RA reservoir strain compared with patients without hepatorenal dysfunction (Table 3). Kaplan–Meier analysis again showed that hepatorenal dysfunction was associated with poor survival (Figure 2A): Survival at 1, 3, and 5 years was 100, 69, and 69%, respectively, in the group with hepatorenal dysfunction, compared with 100, 98, and 98%, respectively, in the rest of the cohort (log-rank *p* = 0.076). Consistent with this, univariate Cox regression showed a significantly increased HR per unit increase of MELDNa (HR: 1.2 [95% CI: 1.0, 1.5], *p* = 0.028). Multivariate Cox regression indicated that hepatorenal dysfunction is associated with survival independent of age, RV systolic function (mirrored by TAPSE), and TR severity (HR: 1.8 [95% CI: 1.1, 3.0], *p* = 0.017). Further adjustment for DOAC intake did not change the results (HR: 1.8 [95% CI: 1.1, 2.9], *p* = 0.013).

In the screening population, follow-up data were available in 91 cases (Supplementary Table 3). A sensitivity analysis of the screening population stratified by the MELD-XI score (24) also showed impaired survival in patients with hepatorenal dysfunction at follow-up (Supplementary Figure 1B; HR: 7.62 [95% CI: 2.23, 26.08], *p* = 0.001).

### 3.5. Improvement of the MELDNa score: a prognostically relevant difference

Finally, the cohort was stratified into three groups according to the change in hepatorenal functional status (based on the MELDNa score) from baseline to follow-up (Figure 2B). The first group presented with a normal MELDNa score at baseline as well as during follow-up (“stable without dysfunction,” *n* = 27), whilst the second group was defined as having hepatorenal dysfunction at baseline, but a low MELDNa score during the follow-up (“improved,” *n* = 7). The third group included those with a high MELDNa score at baseline and during follow-up as well as patients who developed hepatorenal dysfunction during follow-up (“persistent dysfunction or worsening,” *n* = 12).

It became evident that the patients with persistent hepatorenal dysfunction or worsening during follow-up had the poorest prognosis (with survival at 1, 3, and 5 years of 100, 65, and 65%, respectively), whereas none of the patients with steadily normal hepatorenal function died within 5 years after follow-up. Notably, patients who presented with hepatorenal dysfunction at baseline but improved during follow-up showed higher survival (100, 86, and 86% at 1, 3, and 5 years, respectively) than those who had persistent dysfunction or worsening.

## 4. Discussion

To the best of our knowledge, this study is the first to show that hepatorenal dysfunction (assessed *via* the routine lab data-based MELDNa score) is associated with impairment of pulmonary hemodynamics and survival in patients with CTEPH. Venous congestion due to TR and RA engorgement rather than cardiac forward failure is suggested as major link between hemodynamic abnormalities and loss of hepatorenal function.

Hepatorenal dysfunction has been identified as an important prognostic parameter in acute and chronic left heart failure, as well as in congenital heart diseases (6, 15, 26–28). The pathomechanisms behind this close interplay of organs are known to be multifactorial and complex. While heart failure promotes liver disease by ischemia (due to forward failure) as well as congestion (due to backward failure), hepatic dysfunction itself is known to lead to cardiomyocyte apoptosis and cirrhotic cardiomyopathy. This, in turn, accelerates cardiac remodeling, promotes heart failure, and thus increases mortality (29). In the liver, increased venous congestion can lead to congestive hepatopathy, which can cause liver fibrosis and “cirrhose cardiaque” (30). Hepatic vein flow patterns can be used to assess congestive hepatopathy and have been proposed as bedside hepatic dysfunction parameters (10).

Chronic cardiorenal and renocardiac syndromes are characterized by congestive nephropathy, renal hypoperfusion, activation of the renin-angiotensin-aldosterone system and sympathetic nervous system, pro-inflammatory cytokines, and antidiuretic hormone secretion (31), which can then cause further volume retention and cardiac stress (32). Many of these mechanisms have been shown to decrease survival in multiple cardiovascular diseases (33). In this context, intrarenal venous flow patterns and the renal venous stasis index have emerged as feasible echocardiographic parameters with prognostic relevance (11). To sum up, all these mechanisms can lead to a vicious circle in patients with cardiac dysfunction.

Our results suggest that these associations are also applicable for patients with CTEPH. We observed that the MELDNa score predicts mortality in incident as well as during follow-up. Thus, the MELDNa score is a robust and independent predictor of disease severity and survival in CTEPH. Notably, patients whose MELDNa scores improved over time showed substantially higher survival than those with persistent dysfunction or worsening, highlighting the clinical relevance of hepatorenal dysfunction assessed *via* the MELDNa score.

Even though hepatorenal dysfunction is known to be prognostic in other forms of heart failure, the underlying pathomechanism is still not fully understood (29, 32, 34). In addition to forward failure (characterized by impairment of cardiac output), venous congestion and backflow into the vena cava due to tricuspid insufficiency or RA contraction/RV diastolic dysfunction are conceivable. Thus, we sought to determine the driving force behind hepatorenal dysfunction in CTEPH. Interestingly, our study provides evidence that impaired RA function (mirrored by decreased RA reservoir strain) and TR, rather than RV systolic function, are the primary causes of hepatorenal dysfunction. This corresponds to the very early observation of Kussmaul et al. (35), who already described the clinical significance of venous reflux in patients with restricted cardiac filling in 1873. Bilirubin levels were independently associated with RAP in patients with acute heart failure in a more recent study by Biegus et al. (36). Taken together, alterations of the veno–atrial interaction and TR appear to be the underlying causes of organ dysfunction from a pathophysiological point of view.

Although specific normal values or defined cutoffs for the MELDNa score in the context of right heart failure are currently unavailable, the MELDNa score has been utilized as a prognostic marker in various diseases, including triple valve surgery patients and heart failure patients listed for transplant (14, 37). Notably, the observed MELDNa threshold consistently hovered around 10 in these populations. This consistent threshold suggests that a MELDNa score above 10 may indicate a higher risk or severity of hepatorenal dysfunction. While our study focused on CTEPH patients, the utilization of a threshold comparable to those established in other disease contexts enhances the credibility of our findings.

From a clinical perspective, the MELDNa score and its change over time offer a simple way to assess hepatorenal dysfunction and identify patients at risk using easily obtainable laboratory parameters. The clinical management of those patients may then be improved by increasing the frequency of follow-up visits as well as starting treatment escalation, interventional treatment (balloon pulmonary angioplasty), or re-evaluation of eligibility for surgery (pulmonary endarterectomy). In contrast to other risk scores [such as the 2015 European Society of Cardiology/European Respiratory Society risk stratification (38), which has also been shown to predict survival in CTEPH (39, 40)], the simplicity of the MELDNa score allows for routine use worldwide in an ambulatory setting. However, further comparative studies are necessary to determine the most appropriate method of risk stratification in patients with CTEPH.

This study is limited by its retrospective design and missing data. In particular, the follow-up analysis is limited owing to missing data, which may have introduced a bias into the results. The use of the MELDNa score (rather than the MELD-XI score) necessitated the exclusion of 39 patients who used vitamin K antagonists (35% of the 111 patients with MELDNa data) from the primary analyses, which may also have been a source of bias. We chose to focus on the MELDNa score for the primary analyses because of the prognostic importance of hyponatremia (particularly severe hyponatremia) in pulmonary arterial hypertension (41, 42). DOAC use may also affect the MELDNa score. However, use of the INR for the MELDNa calculation may still be possible if the INR is determined at the DOAC trough level (43). As shown in the baseline characteristics, the frequency of DOAC use did not differ between the groups with and without hepatorenal dysfunction, and adjustment for DOAC use did not alter the prognostic significance of the MELDNa score, minimizing potential bias. Consistent with this, the MELD-XI score excluding INR also predicted survival in patients with CTEPH. Patients using drugs possibly affecting liver or kidney function were not excluded from the study, which may affect the results. Nevertheless, we can show that the MELDNa score can be used very well for routine follow-up in real-world patients with CTEPH.

**Figure 3:**
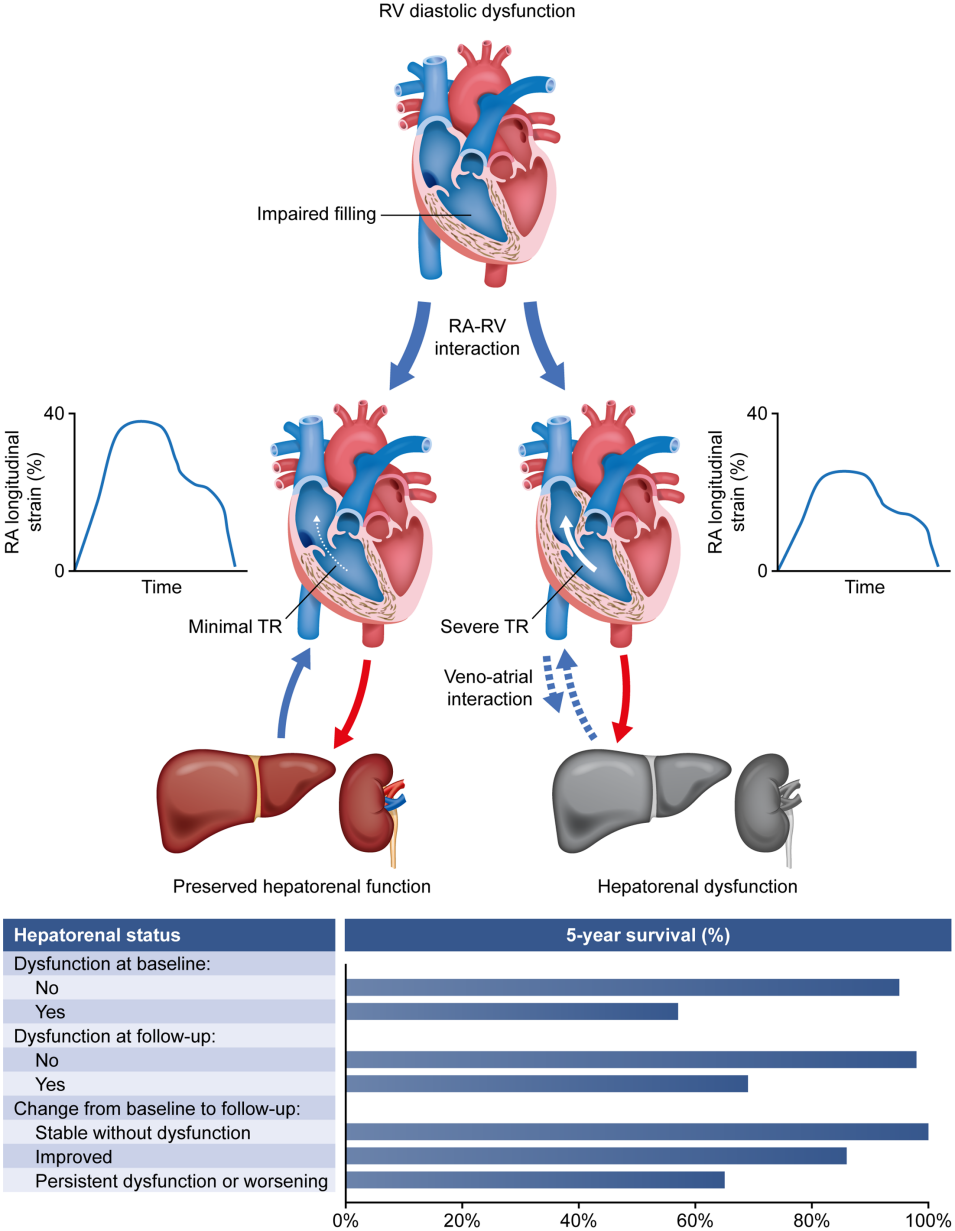
Association of hepatorenal dysfunction with RA dysfunction, TR, and mortality in patients with chronic thromboembolic pulmonary hypertension. RA, right atrial; RV, right ventricular; TR, tricuspid regurgitation.

In conclusion, our data indicate that hepatorenal dysfunction is associated with RA dysfunction and TR, and is a risk factor for mortality when assessed at baseline and during follow-up in patients with CTEPH (Figure 3). Thus, the MELDNa score can help to identify patients at risk. Further prospective studies are required to confirm whether considering the MELDNa score in treatment and risk stratification improves long-term survival in this specific PH population.

## Data availability statement

The raw data supporting the conclusions of this article will be made available by the authors, without undue reservation.

## Ethics statement

The studies involving human participants were reviewed and approved by Local Ethics Committee of Giessen. The patients/participants provided their written informed consent to participate in this study.

## Author contributions

AY, HG, and KT: study conceptualization. AY, DZ, MR, SS, ZR, NK, FG, WS, HoG, HeG, and KT: study design and data collection, critical revision of the manuscript, approval of manuscript for submission, and agreement to be accountable for all aspects of the work in ensuring that questions related to the accuracy or integrity of any part of the work are appropriately investigated and resolved. AY and HG: data analysis. AY, DZ, HG, and KT: drafting of the manuscript. All authors contributed to the article and approved the submitted version.

## Funding

This work was supported by the Excellence Cluster Cardio-Pulmonary System (ECCPS) and the Collaborative Research Center (SFB) 1213—Pulmonary Hypertension and Cor Pulmonale, grant number SFB1213/1, project B08 (German Research Foundation, Bonn, Germany).

## Conflict of interest

AY reports non-financial support from the University of Giessen during the conduct of the study, and personal fees from MSD outside the submitted work. MR reports grants from the German Research Foundation and non-financial support from the University of Giessen during the conduct of the study, and grants from United Therapeutics, grants and personal fees from Bayer, and personal fees from Actelion, Mundipharma, Roche, and OMT outside the submitted work. FG reports no competing interests. WS reports grants from the German Research Foundation and non-financial support from the University of Giessen during the conduct of the study, and personal fees from Pfizer and Bayer Pharma AG outside the submitted work. HAG reports grants from the German Research Foundation and non-financial support from the University of Giessen during the conduct of the study, and personal fees from Bayer, Actelion, Pfizer, Merck, GSK, and Takeda, grants and personal fees from Novartis, Bayer HealthCare, and Encysive/Pfizer, and grants from Aires, the German Research Foundation, Excellence Cluster Cardiopulmonary Research, and the German Ministry for Education and Research outside the submitted work. HG reports grants from the German Research Foundation and non-financial support from the University of Giessen during the conduct of the study, and personal fees from Actelion, AstraZeneca, Bayer, BMS, GSK, Janssen-Cilag, Lilly, MSD, Novartis, OMT, Pfizer, and United Therapeutics outside the submitted work. KT reports grants from the German Research Foundation and non-financial support from the University of Giessen during the conduct of the study, and personal fees from Actelion and Bayer outside the submitted work.

The remaining authors declare that the research was conducted in the absence of any commercial or financial relationships that could be construed as a potential conflict of interest.

## Publisher’s note

All claims expressed in this article are solely those of the authors and do not necessarily represent those of their affiliated organizations, or those of the publisher, the editors and the reviewers. Any product that may be evaluated in this article, or claim that may be made by its manufacturer, is not guaranteed or endorsed by the publisher.

## Abbreviations

BNP: B-type natriuretic peptide
CI: confidence interval
CTEPH: chronic thromboembolic pulmonary hypertension
DOAC: direct oral anticoagulant
GLS: global longitudinal strain
HR: hazard ratio
INR: international normalized ratio
MELDNa: Model for End-stage Liver Disease Sodium
MELD-XI: Model for End-stage Liver Disease Excluding International Normalized Ratio
PH: pulmonary hypertension
RA: right atrial
RAP: right atrial pressure
RV: right ventricular
TAPSE: tricuspid annular plane systolic excursion
TR: tricuspid regurgitation
