# Supplementary material for: Hepatorenal dysfunction in patients with chronic thromboembolic pulmonary hypertension
Source: Front Med (Lausanne). 2023 Jul 20;10:1207474. doi: 10.3389/fmed.2023.1207474 (PMC10399740; doi:10.3389/fmed.2023.1207474)
Supplement: Supplementary file 1 [file Data_Sheet_1.docx]

# Supplementary Figure


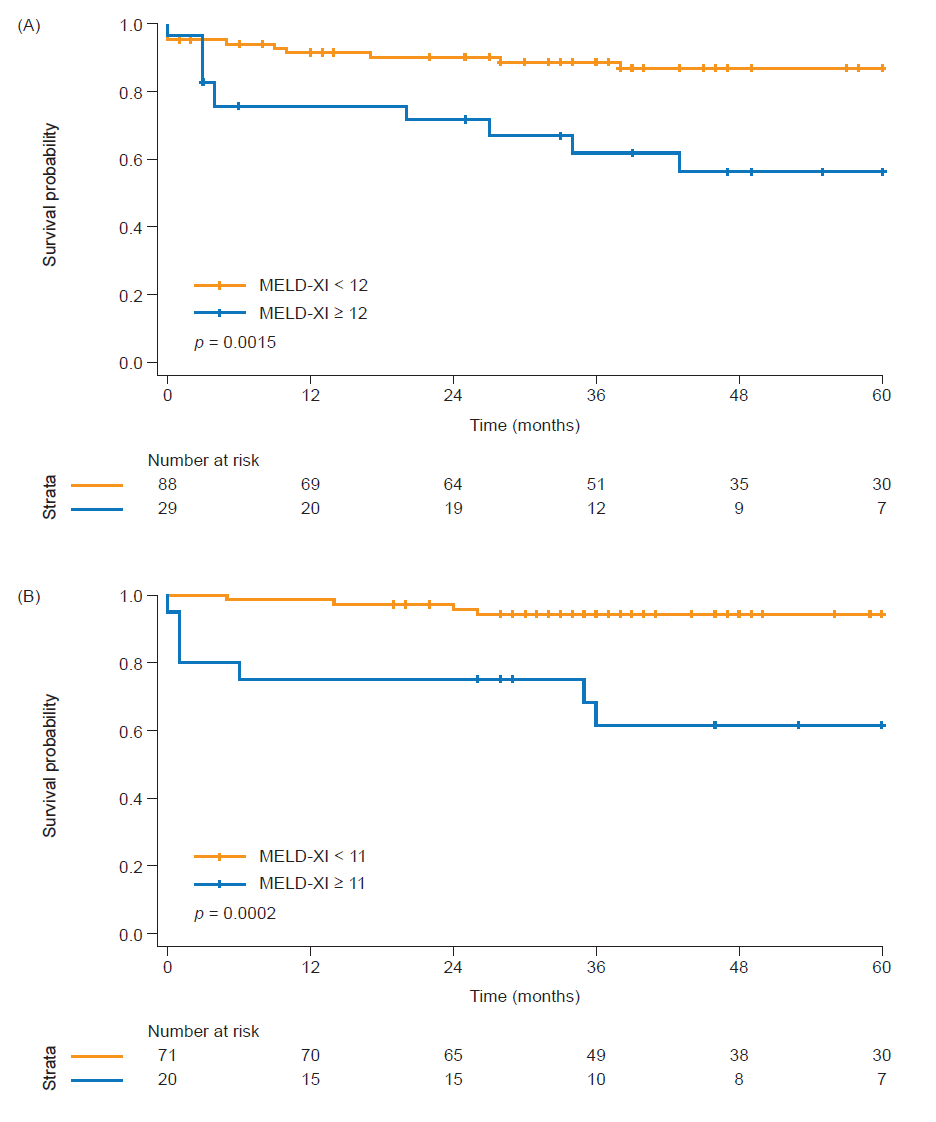


**Supplementary Figure 1. Prognostic role of the MELD-XI score in the screening population.** The MELD-XI score was calculated as 5.11 x ln(bilirubin) + 11.76 x ln(creatinine) + 9.44, as described by Heuman et al. (^[[1]](#endnote-1)^). All screened patients (regardless of vitamin K antagonist use) were included. Patients with a MELD-XI score above the third quartile were compared with the rest of the patients. Kaplan–Meier survival analyses were performed at baseline **(A)** and during follow-up **(B)**. MELD-XI, Model for End-stage Liver Disease Excluding International Normalized Ratio.

# Supplementary Tables

**Supplementary Table 1. Baseline characteristics of the screening population stratified by the MELD-XI score.**

|  | ***n*** | **Preserved hepatorenal function* (*n* = 88)** | **Hepatorenal dysfunction* (*n* = 29)** | **Total  (*n* = 117)** | ***p*** |
| --- | --- | --- | --- | --- | --- |
| Age, years | 116 | 70.0 [55.0, 77.0] | 76.0 [68.0, 80.2] | 72.5 [56.0, 78.0] | 0.052^†^ |
| Male sex, *n* (%) | 117 | 32 (36) | 15 (52) | 47 (40) | 0.143^‡^ |
| Body mass index, kg/m^2^ | 117 | 28.51 [25.40, 32.09] | 27.44 [24.15, 30.10] | 28.01 [25.25, 31.91] | 0.139^†^ |
| NYHA functional class, *n* (%): | 117 |  |  |  | 0.437^‡^ |
| 1 |  | 1 (1) | 1 (3) | 2 (2) |  |
| 2 |  | 8 (9) | 2 (7) | 10 (9) |  |
| 3 |  | 41 (47) | 14 (48) | 55 (47) |  |
| 4 |  | 9 (10) | 6 (21) | 15 (13) |  |
| NA |  | 29 (33) | 6 (21) | 35 (30) |  |
| mPAP, mm Hg | 117 | 38.0 [31.0, 46.2] | 44.0 [36.0, 52.0] | 39.0 [31.0, 47.0] | 0.043^†^ |
| PAWP, mm Hg | 116 | 11.06 ± 4.97 | 11.43 ± 4.70 | 11.15 ± 4.89 | 0.721 |
| RAP, mm Hg | 117 | 7.50 [4.75, 11.00] | 11.00 [8.00, 16.00] | 8.00 [5.00, 11.00] | <0.001^†^ |
| Cardiac output, L/min^§^ | 117 | 4.35 [3.40, 5.14] | 3.80 [2.56, 4.56] | 4.16 [3.30, 5.00] | 0.02^†^ |
| PVR, dyn·s/cm^5^ | 117 | 464 [262, 732] | 727 [473, 914] | 533 [288, 823] | 0.005^†^ |
| Cardiac index, L/min/m^2^ | 117 | 2.470 [2.103, 2.790] | 1.980 [1.800, 2.450] | 2.380 [1.880, 2.750] | 0.006^†^ |
| SvO_2_, % | 117 | 63.2 [59.8, 68.7] | 57.4 [51.4, 64.3] | 62.7 [55.5, 67.7] | 0.013^†^ |
| INR | 117 | 1.100 [1.000, 1.200] | 1.200 [1.000, 1.400] | 1.100 [1.000, 1.200] | 0.011^†^ |
| Sodium, mEq/L | 109 | 140.00 [138.00, 142.00] | 140.00 [137.00, 141.00] | 140.00 [138.00, 141.00] | 0.458^†^ |
| Creatinine, mg/dL | 117 | 0.900 [0.800, 1.125] | 1.300 [1.200, 1.600] | 1.000 [0.800, 1.300] | <0.001^†^ |
| Estimated GFR, mL/min/1.73 m^2^ | 117 | 74.2 [59.3, 92.0] | 42.7 [35.6, 61.5] | 68.6 [49.0, 87.2] | <0.001^†^ |
| Albumin, g/L | 116 | 40.60 [36.75, 43.35] | 39.40 [36.10, 41.60] | 40.50 [36.50, 42.82] | 0.087^†^ |
| Total bilirubin, mg/dL | 117 | 0.600 [0.400, 0.900] | 1.300 [0.900, 1.600] | 0.700 [0.500, 1.200] | <0.001^†^ |
| BNP, pg/mL | 114 | 148.0 [57.2, 315.0] | 475.5 [266.5, 877.2] | 202.0 [77.0, 467.8] | <0.001^†^ |
| TAPSE, mm | 113 | 20.53 ± 3.77 | 17.07 ± 4.14 | 19.68 ± 4.13 | <0.001 |
| S', cm/s | 104 | 12.36 ± 3.59 | 9.31 ± 2.22 | 11.60 ± 3.55 | <0.001 |
| TR severity, *n* (%): | 117 |  |  |  | 0.118^‡^ |
| None |  | 3 (3) | 1 (3) | 4 (3) |  |
| Mild |  | 27 (31) | 2 (7) | 29 (25) |  |
| Moderate |  | 30 (34) | 12 (41) | 42 (36) |  |
| Severe |  | 24 (27) | 11 (38) | 35 (30) |  |
| NA |  | 4 (5) | 3 (10) | 7 (6) |  |
| IVC diameter on expiration, mm | 86 | 19.00 [16.00, 22.00] | 21.00 [19.00, 22.00] | 20.00 [17.00, 22.00] | 0.034^†^ |
| RA reservoir strain, % | 88 | 25.50 [18.16, 32.31] | 14.53 [9.38, 19.80] | 21.27 [14.38, 30.30] | <0.001^†^ |
| RA conduit strain, % | 80 | 8.71 [4.15, 13.13] | 5.00 [2.81, 9.36] | 6.37 [3.48, 13.03] | 0.33^†^ |
| RA active strain, % | 76 | 17.56 ± 6.83 | 9.89 ± 5.67 | 15.74 ± 7.31 | <0.001 |
| RV GLS, % | 58 | −16 [−17.57, −10.6] | −10.55 [−11.62, −8.3] | −13.25 [−17.2, −9.93] | 0.047^†^ |
| RA area, cm^2^ | 105 | 17.10 [13.25, 22.15] | 23.00 [17.40, 26.73] | 18.10 [14.10, 23.50] | 0.003^†^ |
| RV EDD, cm | 105 | 4.825 ± 0.830 | 5.174 ± 1.029 | 4.912 ± 0.891 | 0.125 |
| MELDNa score | 109 | 9.14 ± 3.43 | 14.13 ± 4.33 | 10.47 ± 4.29 |  |
|  |  | 8.95 [6.36, 11.36] | 13.64 [11.95, 15.61] | 10.18 [7.50, 13.14] | <0.001^†^ |
| DOAC, *n* (%) | 117 |  |  |  | 0.049^‡^ |
| No |  | 32 (36) | 18 (62) | 50 (43) |  |
| Yes |  | 55 (62) | 11 (38) | 66 (56) |  |
| NA |  | 1 (1) | 0 | 1 (1) |  |
|  |  |  |  |  |  |

Data are presented as mean ± standard deviation, median [interquartile range], or *n* (%). *P* values are from t-tests unless otherwise specified. *Hepatorenal dysfunction was defined as a MELD-XI score ≥ 12 (above the third quartile). ^†^Wilcoxon test. ^‡^Pearson test for categorical variables. ^§^Measured by thermodilution. BNP, B-type natriuretic peptide; DOAC, direct oral anticoagulant; EDD, end-diastolic diameter; GFR, glomerular filtration rate; GLS, global longitudinal strain; INR, International Normalized Ratio; IVC, inferior vena cava; MELDNa, Model for End-stage Liver Disease Sodium; MELD-XI, Model for End-stage Liver Disease Sodium Excluding International Normalized Ratio; mPAP, mean pulmonary arterial pressure; NA, not available; NYHA FC, New York Heart Association functional class; PAWP, pulmonary arterial wedge pressure; PVR, pulmonary vascular resistance; RA, right atrial; RAP, right atrial pressure; RV, right ventricular; SvO_2_, mixed venous oxygen saturation; TAPSE, tricuspid annular plane systolic excursion; TR, tricuspid regurgitation.

**Supplementary Table 2. Baseline characteristics of the cohort with available MELDNa scores (including patients taking vitamin K antagonists).**

|  | ***n*** | **Preserved hepatorenal function* (*n* = 65)** | **Hepatorenal dysfunction* (*n* = 46)** | **Total  (*n* = 111)** | ***p*** |
| --- | --- | --- | --- | --- | --- |
| Age, years | 110 | 74.0 [55.0, 78.0] | 72.0 [56.0, 79.0] | 73.0 [55.2, 78.0] | 0.906^†^ |
| Male sex, *n* (%) | 111 | 22 (34) | 23 (50) | 45 (41) | 0.088^‡^ |
| Body mass index, kg/m^2^ | 111 | 27.64 [25.56, 31.40] | 28.57 [24.27, 33.94] | 28.08 [25.31, 31.98] | 1^†^ |
| NYHA functional class, *n* (%): | 111 |  |  |  | 0.58^‡^ |
| 1 |  | 1 (2) | 1 (2) | 2 (2) |  |
| 2 |  | 7 (11) | 4 (9) | 11 (10) |  |
| 3 |  | 30 (46) | 21 (46) | 51 (46) |  |
| 4 |  | 5 (8) | 8 (17) | 13 (12) |  |
| NA |  | 22 (34) | 12 (26) | 34 (31) |  |
| mPAP, mm Hg | 111 | 36.0 [28.0, 47.0] | 41.5 [36.2, 47.0] | 38.0 [31.0, 47.0] | 0.007^†^ |
| PAWP, mm Hg | 110 | 10.75 ± 4.32 | 11.89 ± 5.86 | 11.22 ± 5.01 | 0.271 |
| RAP, mm Hg | 111 | 7.00 [5.00, 10.00] | 11.00 [7.00, 15.75] | 8.00 [5.00, 11.00] | 0.002^†^ |
| Cardiac output, L/min^§^ | 111 | 4.33 [3.43, 5.13] | 3.93 [2.90, 4.76] | 4.16 [3.18, 5.04] | 0.085^†^ |
| PVR, dyn·s/cm^5^ | 111 | 400 [242, 720] | 574 [390, 872] | 504 [271, 811] | 0.013^†^ |
| Cardiac index, L/min/m^2^ | 111 | 2.530 [2.060, 2.890] | 2.240 [1.805, 2.553] | 2.400 [1.875, 2.755] | 0.01^†^ |
| SvO_2_, % | 111 | 64.20 [60.40, 69.40] | 57.35 [50.75, 63.25] | 62.60 [55.45, 67.65] | <0.001^†^ |
| INR | 111 | 1.100 [1.000, 1.100] | 1.200 [1.100, 1.500] | 1.100 [1.000, 1.300] | <0.001^†^ |
| Sodium, mEq/L | 111 | 141.00 [139.00, 142.00] | 138.00 [136.25, 140.75] | 140.00 [138.00, 141.00] | <0.001^†^ |
| Creatinine, mg/dL | 111 | 0.900 [0.800, 1.100] | 1.300 [1.000, 1.600] | 1.000 [0.800, 1.300] | <0.001^†^ |
| Estimated GFR, mL/min/1.73 m^2^ | 111 | 74.1 [63.9, 88.2] | 51.5 [35.9, 71.2] | 68.6 [47.0, 86.5] | <0.001^†^ |
| Albumin, g/L | 110 | 41.60 [38.32, 43.50] | 39.25 [35.40, 41.58] | 40.60 [36.52, 42.90] | 0.005^†^ |
| Total bilirubin, mg/dL | 111 | 0.600 [0.400, 0.900] | 1.000 [0.600, 1.400] | 0.700 [0.500, 1.200] | <0.001^†^ |
| BNP, pg/mL | 108 | 117.5 [48.0, 254.2] | 475.5 [198.5, 842.8] | 209.0 [74.8, 474.2] | <0.001^†^ |
| TAPSE, mm | 107 | 20.76 ± 3.62 | 17.80 ± 4.44 | 19.57 ± 4.21 | <0.001 |
| S', cm/s | 99 | 12.41 ± 3.73 | 10.50 ± 3.27 | 11.60 ± 3.65 | 0.008 |
| TR severity, *n* (%): | 111 |  |  |  | 0.004^‡^ |
| None |  | 2 (3) | 2 (4) | 4 (4) |  |
| Mild |  | 23 (35) | 5 (11) | 28 (25) |  |
| Moderate |  | 26 (40) | 15 (33) | 41 (37) |  |
| Severe |  | 13 (20) | 19 (41) | 32 (29) |  |
| NA |  | 1 (2) | 5 (11) | 6 (5) |  |
| IVC diameter on expiration, mm | 81 | 19.00 [15.00, 22.00] | 21.00 [18.00, 24.00] | 20.00 [17.00, 22.00] | 0.012^†^ |
| RA reservoir strain, % | 82 | 27.8 [18.6, 33.4] | 15.9 [12.3, 22.2] | 20.5 [14.3, 30.0] | <0.001^†^ |
| RA conduit strain, % | 75 | 9.06 [4.34, 13.49] | 5.35 [3.03, 10.05] | 6.19 [3.47, 12.79] | 0.112^†^ |
| RA active strain, % | 71 | 17.76 ± 6.95 | 12.10 ± 7.30 | 15.37 ± 7.59 | 0.002 |
| RV GLS, % | 56 | −16.40 [−19.50, −9.80] | −11.00 [−14.75, −9.20] | −12.90 [−17.20, −9.58] | 0.024^†^ |
| RA area, cm^2^ | 99 | 16.20 [12.88, 21.73] | 20.90 [17.30, 25.40] | 18.10 [14.20, 23.60] | <0.001^†^ |
| RV EDD, cm | 99 | 4.646 ± 0.871 | 5.260 ± 0.813 | 4.906 ± 0.896 | <0.001 |
| MELDNa score | 111 | 7.62 ± 2.21 | 14.75 ± 3.35 | 10.58 ± 4.46 |  |
|  |  | 7.84 [5.87, 9.26] | 13.95 [12.77, 15.72] | 10.18 [7.56, 13.17] | <0.001^†^ |
| DOAC, *n* (%) | 111 |  |  |  | 0.004^‡^ |
| No |  | 21 (32) | 28 (61) | 49 (44) |  |
| Yes |  | 44 (68) | 17 (37) | 61 (55) |  |
| NA |  | 0 | 1 (2) | 1 (1) |  |
|  |  |  |  |  |  |

Data are presented as mean ± standard deviation, median [interquartile range], or *n* (%). *P* values are from t-tests unless otherwise specified. *Hepatorenal dysfunction was defined as a MELDNa score > 11 (above the third quartile in the subset not using vitamin K antagonist treatment [primary analysis population]). ^†^Wilcoxon test. ^‡^Pearson test for categorical variables. ^§^Measured by thermodilution. BNP, B-type natriuretic peptide; DOAC, direct oral anticoagulant; EDD, end-diastolic diameter; GFR, glomerular filtration rate; GLS, global longitudinal strain; INR, International Normalized Ratio; IVC, inferior vena cava; MELDNa, Model for End-stage Liver Disease Sodium; mPAP, mean pulmonary arterial pressure; NA, not available; NYHA FC, New York Heart Association functional class; PAWP, pulmonary arterial wedge pressure; PVR, pulmonary vascular resistance; RA, right atrial; RAP, right atrial pressure; RV, right ventricular; SvO_2_, mixed venous oxygen saturation; TAPSE, tricuspid annular plane systolic excursion; TR, tricuspid regurgitation.**Supplementary Table 3. Characteristics of the screening population at follow-up stratified by the MELD-XI score.**

|  | ***n*** | **Preserved hepatorenal function* (*n* = 71)** | **Hepatorenal dysfunction* (*n* = 20)** | **Total  (*n* = 91)** | ***p*** |
| --- | --- | --- | --- | --- | --- |
| Age, years | 90 | 69.0 [57.0, 77.0] | 73.0 [60.5, 83.0] | 69.5 [57.0, 78.0] | 0.51^†^ |
| Male sex, *n* (%) | 91 | 26 (37) | 13 (65) | 39 (43) | 0.023^‡^ |
| Body mass index, kg/m^2^ | 70 | 27.45 [25.19, 31.23] | 26.37 [23.20, 29.78] | 27.44 [24.83, 30.96] | 0.515^†^ |
| NYHA functional class, *n* (%): | 91 |  |  |  | 0.407^‡^ |
| 2 |  | 17 (24) | 2 (10) | 19 (21) |  |
| 3 |  | 32 (45) | 9 (45) | 41 (45) |  |
| 4 |  | 1 (1) | 1 (5) | 2 (2) |  |
| NA |  | 21 (30) | 8 (40) | 29 (32) |  |
| mPAP, mm Hg | 43 | 32.00 [23.00, 39.00] | 41.50 [38.50, 45.50] | 34.00 [26.00, 40.50] | 0.009^†^ |
| PAWP, mm Hg | 43 | 11.29 ± 4.46 | 8.75 ± 4.53 | 10.81 ± 4.53 | 0.181 |
| RAP, mm Hg | 43 | 7.00 [3.00, 10.00] | 7.00 [6.00, 7.75] | 7.00 [3.00, 10.00] | 0.829^†^ |
| Cardiac output, L/min^§^ | 43 | 4.99 [4.30, 5.88] | 4.16 [3.13, 5.72] | 4.95 [4.19, 5.88] | 0.188^†^ |
| PVR, dyn·s/cm^5^ | 43 | 254 [193, 425] | 636 [450, 810] | 307 [203, 496] | <0.001^†^ |
| Cardiac index, L/min/m^2^ | 43 | 2.860 [2.435, 3.270] | 2.230 [1.895, 2.438] | 2.750 [2.355, 3.210] | 0.002^†^ |
| SvO_2_, % | 43 | 65.90 [61.90, 70.35] | 59.80 [57.33, 62.87] | 64.20 [59.80, 69.50] | 0.011^†^ |
| INR | 90 | 1.100 [1.000, 1.500] | 1.450 [1.175, 2.525] | 1.100 [1.000, 1.750] | 0.003^†^ |
| Sodium, mEq/L | 90 | 140.00 [138.00, 141.75] | 137.50 [134.75, 141.00] | 139.50 [137.25, 141.00] | 0.051^†^ |
| Creatinine, mg/dL | 91 | 0.900 [0.700, 1.100] | 1.500 [1.200, 1.875] | 1.000 [0.800, 1.200] | <0.001^†^ |
| Estimated GFR, mL/min/1.73 m^2^ | 91 | 75.2 [60.1, 93.8] | 45.0 [32.2, 56.9] | 65.6 [50.6, 88.1] | <0.001^†^ |
| Albumin, g/L | 90 | 42.40 [39.82, 44.32] | 41.75 [40.70, 44.28] | 42.30 [39.92, 44.35] | 0.836^†^ |
| Total bilirubin, mg/dL | 91 | 0.600 [0.400, 0.750] | 1.300 [0.750, 1.600] | 0.600 [0.400, 0.950] | <0.001^†^ |
| BNP, pg/mL | 87 | 96.0 [42.5, 177.5] | 430.0 [117.5, 836.5] | 116.0 [52.0, 314.0] | <0.001^†^ |
| TAPSE, mm | 90 | 21.00 ± 4.16 | 18.62 ± 4.84 | 20.47 ± 4.40 | 0.056 |
| S', cm/s | 86 | 12.70 ± 2.87 | 11.53 ± 4.18 | 12.44 ± 3.21 | 0.262 |
| TR severity, *n* (%): | 91 |  |  |  | 0.406^‡^ |
| None |  | 2 (3) | 1 (5) | 3 (3) |  |
| Mild |  | 29 (41) | 4 (20) | 33 (36) |  |
| Moderate |  | 22 (31) | 8 (40) | 30 (33) |  |
| Severe |  | 16 (23) | 7 (35) | 23 (25) |  |
| NA |  | 2 (3) | 0 | 2 (2) |  |
| IVC diameter on expiration, mm | 74 | 18.00 [16.00, 20.00] | 19.00 [18.00, 24.00] | 18.00 [16.00, 20.00] | 0.109^†^ |
| RA reservoir strain, % | 68 | 27.00 [21.58, 34.56] | 13.59 [9.22, 21.25] | 25.44 [18.40, 32.10] | <0.001^†^ |
| RV GLS, % | 50 | −16.9 [−20.75, −14.55] | −12.7 [−15.6, −10.1] | −16.2 [−20.05, −13.25] | 0.004^†^ |
| RA area, cm^2^ | 82 | 16.30 [14.20, 19.60] | 21.50 [17.40, 25.50] | 16.90 [14.75, 20.95] | 0.003^†^ |
| RV EDD, cm | 82 | 4.722 ± 0.688 | 4.929 ± 0.790 | 4.765 ± 0.710 | 0.332 |
| MELDNa score | 90 | 9.12 [6.71, 13.24] | 19.10 [13.74, 22.64] | 10.15 [7.50, 16.67] | <0.001^†^ |
|  |  | 10.22 ± 5.41 | 19.39 ± 7.32 | 12.26 ± 6.99 |  |
| DOAC, *n* (%) | 91 |  |  |  | 0.058^‡^ |
| No |  | 17 (24) | 9 (45) | 26 (29) |  |
| Yes |  | 46 (65) | 7 (35) | 53 (58) |  |
| NA |  | 8 (11) | 4 (20) | 12 (13) |  |
| Riociguat, *n* (%) | 91 |  |  |  | 0.462^‡^ |
| No |  | 26 (37) | 5 (25) | 31 (34) |  |
| Yes |  | 37 (52) | 11 (55) | 48 (53) |  |
| NA |  | 8 (11) | 4 (20) | 12 (13) |  |
| PEA up to follow-up, *n* (%) | 91 |  |  |  | 0.019^‡^ |
| No |  | 54 (76) | 20 (100) | 74 (81) |  |
| Yes |  | 17 (24) | 0 | 17 (19) |  |
| BPA up to follow-up, *n* (%) | 91 |  |  |  | 0.245^‡^ |
| No |  | 69 (97) | 20 (100) | 89 (98) |  |
| Yes |  | 2 (3) | 0 | 2 (2) |  |
|  |  |  |  |  |  |

Data are presented as mean ± standard deviation, median [interquartile range], or *n* (%). *P* values are from t-tests unless otherwise specified. *Hepatorenal dysfunction was defined as a MELD-XI score ≥ 11 (above the third quartile). ^†^Wilcoxon test. ^‡^Pearson test for categorical variables. ^§^Measured by thermodilution. BNP, B-type natriuretic peptide; BPA, balloon pulmonary angioplasty; DOAC, direct oral anticoagulant; EDD, end-diastolic diameter; GFR, glomerular filtration rate; GLS, global longitudinal strain; INR, International Normalized Ratio; IVC, inferior vena cava; MELDNa, Model for End-stage Liver Disease Sodium; MELD-XI, Model for End-stage Liver Disease Sodium Excluding International Normalized Ratio; mPAP, mean pulmonary arterial pressure; NA, not available; NYHA FC, New York Heart Association functional class; PAWP, pulmonary arterial wedge pressure; PEA, pulmonary endarterectomy; PVR, pulmonary vascular resistance; RA, right atrial; RAP, right atrial pressure; RV, right ventricular; SvO_2_, mixed venous oxygen saturation; TAPSE, tricuspid annular plane systolic excursion; TR, tricuspid regurgitation.

# Supplementary reference

1. Heuman DM, Mihas AA, Habib A, Gilles HS, Stravitz RT, Sanyal AJ, Fisher RA. MELD-XI: a rational approach to "sickest first" liver transplantation in cirrhotic patients requiring anticoagulant therapy. Liver Transpl. 2007 Jan;13(1):30-7. doi: 10.1002/lt.20906. Epub 2006 Dec 13. PMID: 17154400. [↑](#endnote-ref-1)
